# Rapid destruction of shoulder joint by pigmented villonodular synovitis treated by hemiarthroplasty: A case report

**DOI:** 10.1016/j.ijscr.2020.10.128

**Published:** 2020-11-01

**Authors:** Minsung Kwon, Jin-Young Bang, Kyung Han Nam

**Affiliations:** a Department of Orthopaedic Surgery, Inje University College of Medicine, Haeundae Paik Hospital, 875 Haeundae-ro, Haeundae-gu, Busan, 48108, Republic of Korea; b Department of Pathology, Inje University College of Medicine, Haeundae Paik Hospital, 875 Haeundae-ro, Haeundae-gu, Busan, 48108, Republic of Korea

**Keywords:** Pigmented villous nodular synovitis, Shoulder, Arthroplasty, Hemiarthroplasty, Arthritis, Giant cell

## Abstract

**Introduction:**

Pigmented villous nodular synovitis is an uncommon proliferative disease of the joints, and rarely reported in the shoulder. It can become symptomatic when proliferating soft tissue infiltrates a joint, causing arthritic changes including bone erosion. The literature has described the disease progression as indolent. Here we report on a case of PVNS of the shoulder joint which rapidly lead to significant bone damage and was subsequently treated by shoulder arthroplasty.

**Presentation of case:**

We report here on a 71-year old female patient who presented with a 6 month history of aggravating shoulder pain. Radiography imaging over a one month period indicated rapid joint destruction. Magnetic resonance imaging suggested the presence of PVNS of the shoulder joint and significant bone erosion. The patient was subsequently treated by shoulder arthroplasty performed by authors. Histological examination confirmed the PVNS diagnosis. Shoulder pain significantly decreased during the follow up period, and the patient was able to resume daily activities.

**Discussion:**

Comparing to Milwaukee shoulder syndrome or a joint infection, PVNS is known to progress indolently. However, our case clearly showed that PVNS could also cause radical destruction of the joint. Previous reports showed the high recurrence rate of PVNS after joint preserving surgery. In our experience hemiarthroplasty could be the choice of treatment with the low recurrence rate and high functional outcome.

**Conclusions:**

Physicians should include PVNS in the differential diagnosis when they are presented with evidence of rapid destruction of the shoulder joint. Hemiarthroplasty could be treatment option for PVNS of shoulder joint.

## Highlights

• PVNS could progress rapidly causing devastating arthritic changes in the joint.
• Clinicians should also consider PVNS as a reason for shoulder destruction.
• Hemiarthroplasty could be treatment option for PVNS of shoulder joint.

## 1 Introduction

Pigmented villous nodular synovitis (PVNS) is a relatively rare disease. The incision of PVNS is 1.8 cases per 1 million people per year [1]. PVNS most commonly occurs in the fourth and fifth decades of life, and only 2.4% of cases involve the shoulder joint [2]. Previous studies reported that the disease could invade the joints, resulting in sequelae including arthritic changes to the joint. Progression of the disease, however, is generally described as indolent [3]. To our best knowledge, rapid progression of PVNS-induced damage to the shoulder joint has not been reported in the literature. We report a case of PVNS of the shoulder joint that aggravated rapidly and was treated by shoulder arthroplasty.

## 2 Presentation of case

A 71-year-old woman presented with a 6-month history of aggravating right shoulder pain. The patient was a hepatitis C virus carrier but had no family history of any disease. Conservative treatment including nonsteroidal anti-inflammatory drugs and a onetime steroid injection did not relieve the shoulder pain. On orthopedic physical examination, mild joint swelling with a decreased range of motion due to pain was observed. Neither warmth nor redness of the shoulder was observed upon physical examination. Laboratory results were unremarkable, indicating no sign of joint infection or any other joint disorder: -white blood cell count: 5.11 × 10^9/L (neutrophil 42.8%) (normal range 4.0–10.0 × 10^9/L), C-reactive protein level: 0.22 mg/dl (normal range >0.30 mg/dl), and uric acid level: 4.1 mg/dl (normal range 3.0–5.5 mg/dl).

**Fig. 1:**
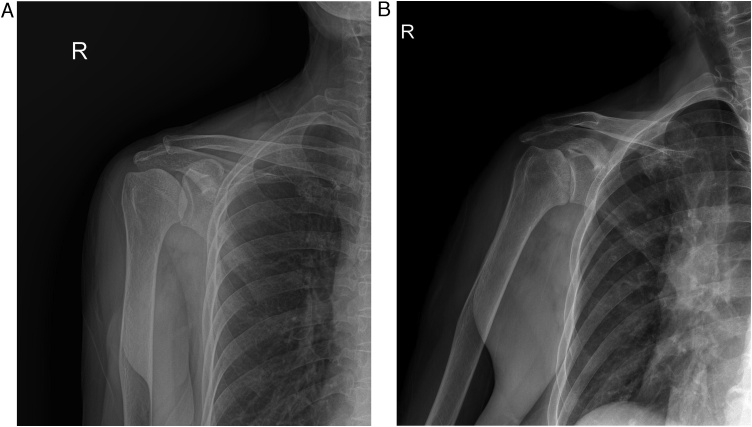
Right shoulder plain anteroposterior (AP) radiograph (a). Rapid progression of joint destruction seen at the 4-week follow-up in the AP view (b).

A comparison of plain radiographs obtained at the initial presentation and at the 1-month follow-up visit demonstrated rapid progression of joint degradation, resulting in loss of humerus sphericity and narrowing of the joint space (Fig. 1a and b).

**Fig. 2:**
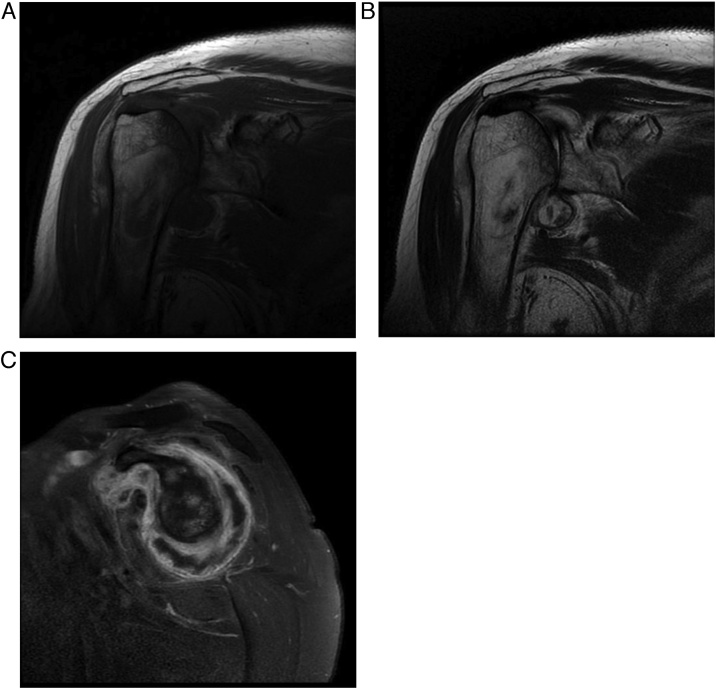
Right shoulder magnetic resonance image, T1-weighted image showing a low signal intensity mass-like lesion (a). T2-weighted image showing low signal change, suggesting presence of hemosiderin deposit (b). Enhanced view showing enhancement of the mass-like lesion (c).

Magnetic resonance imaging showed broad cartilage destruction with joint swelling. Synovial proliferation and enhancement of both the right glenohumeral joint and subcoracoid bursal space were noted, as was proliferation of the biceps brachii long head tendon sheath. Low signal change in T1- and T2-weighted images suggested PVNS of the shoulder joint as low signal change in T2-weighted image suggests the presence of hemosiderin deposits. The glenoid cartilage was preserved, and no distal invasion of PVNS was observed (Fig. 2a–c).

**Fig. 3:**
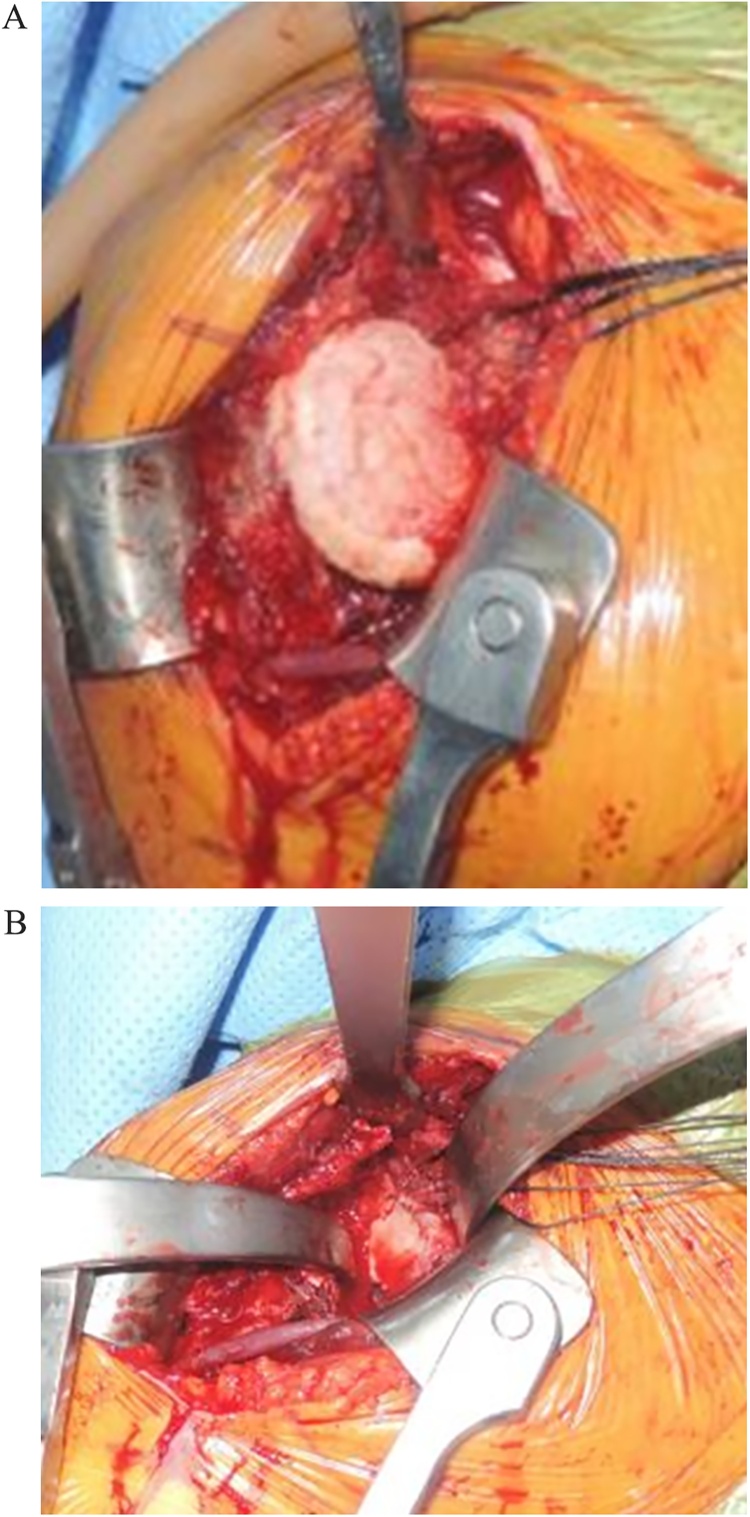
Intraoperative images. Collapsed humerus head (a). Preservation of glenoid cartilage (b).

**Fig. 4:**
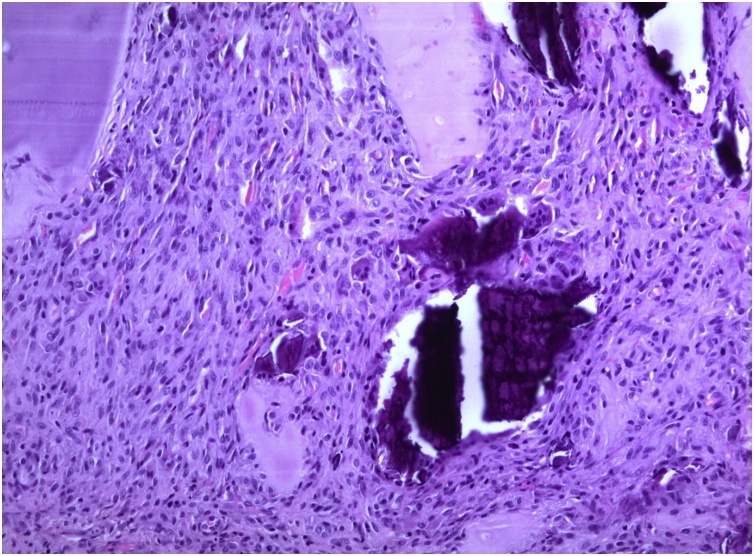
Histopathological analysis at 100× magnification. The arrow indicates a multinucleated giant cell.

**Fig. 5:**
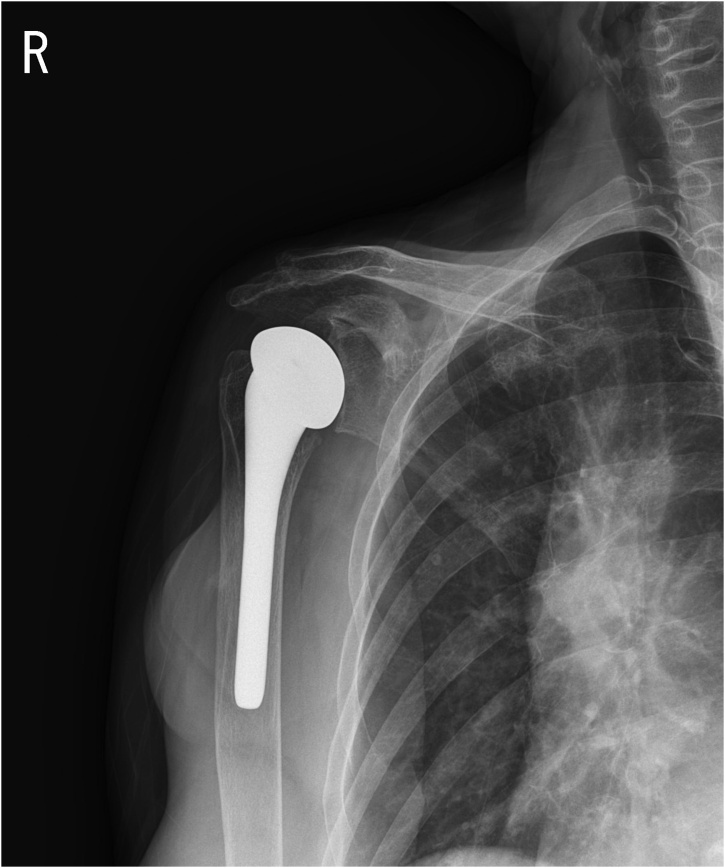
7-months postoperative follow-up plain AP view.

Shoulder hemiarthroplasty was performed by authors using a deltopectoral approach. Intraoperative findings clearly showed arthritic changes of the humerus. The humeral head collapsed, but the cartilage of glenoid remained intact (Fig. 3a and b). A brownish nodular synovium was found, which highly suggested PVNS. Histological examination of the excised synovium indicated synovium proliferation with hemosiderin deposits (Fig. 4). The presence of giant cells and mononuclear cells also implied that the mass was PVNS. After surgery, the patient used a shoulder abduction brace for 6 weeks, and she was allowed to perform daily activities without restriction. The patient showed significant decrease in shoulder pain during the follow-up period and satisfied with the treatment. Last plain radiography was performed at 7 months after surgery. Patient showed significant restoration of range of motion from 40° abduction, 10° forward flexion to 120° abduction with 50° forward flexion further flexion (Fig. 5). Written informed consent was obtained from the patient during the hospitalization period.

## 3 Discussion

Intra-articular PVNS in the shoulder joint is uncommon. PVNS of the large joints most commonly involves the knee (66%, of cases), followed by the hip and the ankle [4,5]. Only 2.4% of PVNS invades the shoulder joint [2,6], and most patients remain asymptomatic for years without damage to the joint. Treatment of symptomatic PVNS often requires the excision of the mass and associated soft tissue. However, PVNS has a high recurrence rate, approximately 46%, following surgical excision [7]. Because PVNS is thought to be a slowly progressing disease, orthopedic physicians are more likely to consider Milwaukee shoulder syndrome, osteonecrosis of humeral head or a joint infection when confronted with a rapidly destructive shoulder disease [9]. This case history suggests that PVNS could also cause radical destruction of the joint. Considering the high recurrence rate of PVNS after conventional arthroscopic resection or open resection, arthroplasty should also be considered for elderly patients with arthritic changes such as those observed in this study. If glenoid component is also affected by PVNS, total or reverse total shoulder arthroplasty should be considered [10].

This work has been reported in line with the SCARE 2018 criteria [8].

## 4 Conclusion

PVNS is a relatively rare disease known to be indolent. However, it could progress rapidly causing devastating arthritic changes in the joint, as observed in our case. Although Milwaukee shoulder syndrome is known to be the most common cause of rapidly destructive shoulder disease, clinicians should also consider PVNS as a reason for shoulder destruction.

## Declaration of Competing Interest

The authors report no declarations of interest.

## Funding

This research received no specific grant from any funding agency in the public, commercial, or not-for-profit sectors.

## Ethical approval

Our institution does not require ethical approval for reporting individual cases or case series.

## Consent

Written informed consent was obtained from the patient(s) for their anonymized information to be published in this article.

## Author’s contribution

Minsung Kwon wrote the manuscript.

Jin-Young Bang determined the research design.

Kyung Han Nam analysed pathologic findings.

## Registration of research studies

1. Name of the registry: Researchregistry.
2. Unique identifying number or registration ID: researchregistry5946.
3. Hyperlink to your specific registration (must be publicly accessible and will be checked): https://www.researchregistry.com/browse-the-registry#home/registrationdetails/5f4667987eb52b001969f01a/.

## Guarantor

Jin-Young Bang.

## Provenance and peer review

Not commissioned, externally peer-reviewed.
